# Clinical Neuropsychology in Germany

**DOI:** 10.3389/fpsyg.2022.843319

**Published:** 2022-02-28

**Authors:** Erich Kasten

**Affiliations:** Medical School Hamburg, Hamburg, Germany

**Keywords:** neuropsychology, therapy brain lesion, CNS-damage treatment, Germany, health assurance system

## Abstract

Clinical neuropsychologists have been working in Germany since the 1980s, and specific training in the discipline has been available since 1993. The qualification currently requires 3 years of practical training, 400 h of theoretical learning, 100 h of supervision, five reports on patients and an oral examination. After its completion, neuropsychologists can work as employees in clinical settings. For a substantial period of time, neuropsychologists working in their own practices faced complex challenges in working with outpatients, whose health insurers did not cover the cost of this treatment. State approval of neuropsychological diagnostic procedures and therapy was achieved in 2011, on the basis of evidence showing the method’s high effectiveness; statutory health insurers therefore now pay out for delivery of these services on an outpatient basis, too. In Germany, neuropsychologists work in all areas of the diagnosis of functional disorders, with children, adolescents, adults and older adults, and carry out treatment. Clinical neuropsychologists provide patients with individualized tools for managing their brain damage, supply exercises and tasks for them to undertake at home, and give input on administrative matters, such as determining the degree of a patient’s disability. Treatment takes place in close collaboration with members of related professions, such as physiotherapists, occupational therapists and speech therapists. Neuropsychologists also work as assessors, investigating, for example, the extent of a patient’s ability to work after sustaining CNS damage or the need for medical retirement. The earnings of clinical neuropsychologists vary widely, currently averaging between €3,000 and €4,000 gross per month for employed neuropsychologists; self-employed neuropsychologists in their own practices can currently bill statutory health insurers for around €100.00 per hour. Despite the generally good working conditions in this discipline, Germany is suffering from a shortage of clinical neuropsychologists. An approximate total of 800 psychologists have completed training in this field, while around 50,000 patients could benefit from neuropsychological treatment every year.

## The History of Neuropsychology in Germany

The neurosciences have a long tradition in Germany. The neurologist Alois Alzheimer (1864–1915), the anatomist Korbinian Brodmann (1868–1918), Hans-Gerhard Creutzfeld (1885–1964), Paul Julius Moebius (1853–1907), and the neurologist Carl Wernicke (1848–1905), who gave his name to Wernicke’s aphasia, are some examples of well-known scientists in this field. Karl-August Weinhold (1782–1828), a professor of surgery who regarded the brain essentially as a generator of electricity, achieved rather macabre fame in this area when—as he describes in his book—he removed the brain of a kitten and filled the spinal opening with zinc and silver, two metals capable of producing electricity, whereupon, in his account, the young cat came back to life and began to jump around (Weinhold, 1817; Finger and Law, 1998).

**Figure 1:**
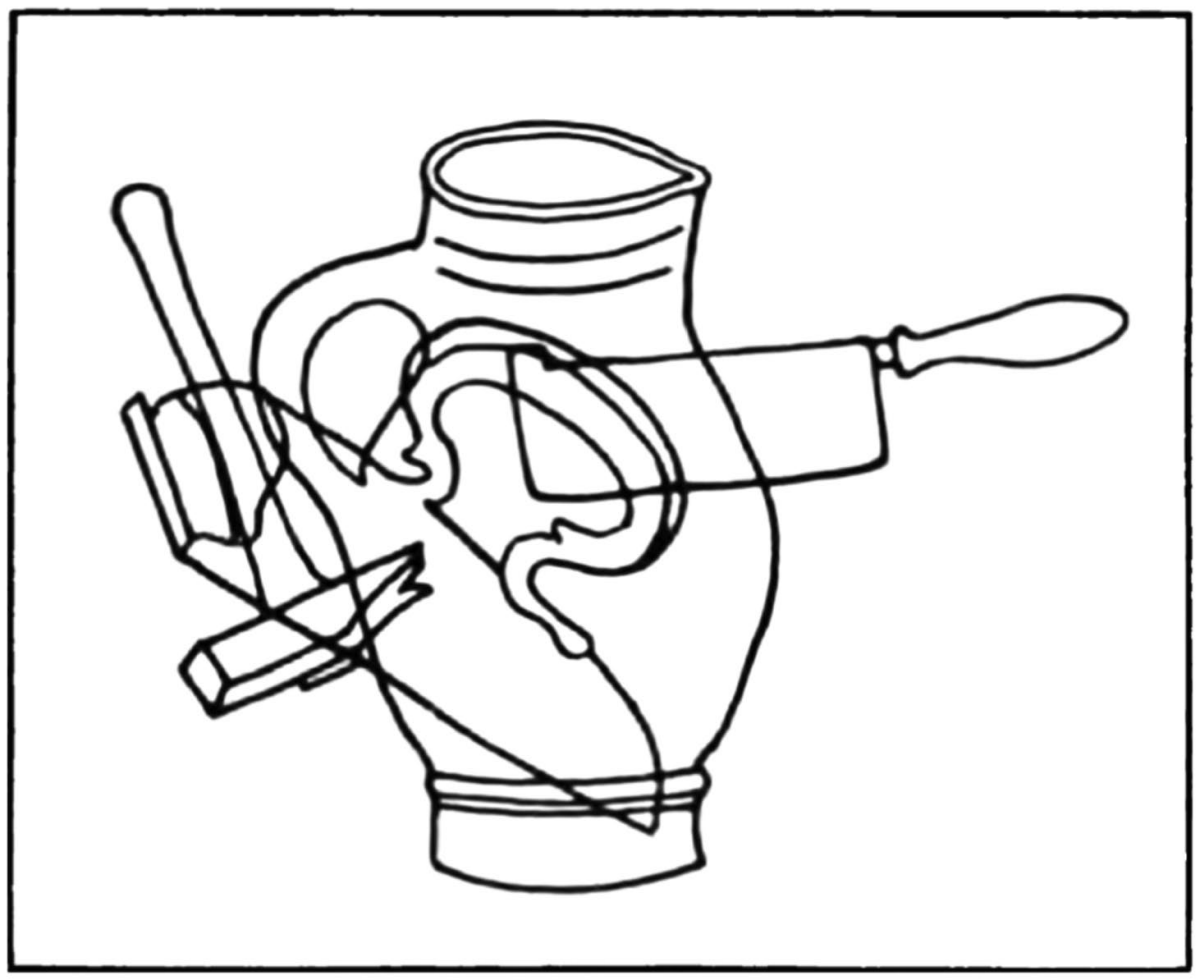
Poppelreuter’s test for examining visual disorders.

As early as 1915, Kurt Goldstein and Ademar Gelb founded a center in Frankfurt am Main for the rehabilitation of soldiers with brain injuries (Dick et al., 1995). One of the most important early representatives of clinical neuropsychology in Germany was doubtless Walther Poppelreuter (1886–1939), who treated First World War veterans with brain injuries, developing a wide range of psychometric examination and treatment methods for this purpose. The Poppelreuter figure (Figure 1), which he designed, is still in use today, in various modifications, for testing visual perceptual function. More than 100 years ago, Poppelreuter pointed out that the symptoms of brain injuries fell into three categories: (1) the subjective complex of brain damage, (2) an objective reduction in physical and mental capacity, and (3) a change in the patient’s overall personality (Poppelreuter, 1918, 1929). Historically, it may be interesting that Kurt Goldstein was Jewish, in 1933 he was arrested during the era of Hitler’s Reich, sent to a concentration camp and was forced to emigrate, while Walther Poppelreuter was a supporter of the German Nazi regime and even wrote a book in 1934 entitled “Hitler, the political psychologist.”

One of the first pioneering German textbooks in this area was the volume *Neuropsychologische Rehabilitation: Grundlagen—Diagnostik—Behandlungsmethoden* (Neuropsychological Rehabilitation: Foundations—Diagnostics—Treatment Methods), published in 1988 by von Cramon and Zihl (1988). At this time, the neurosciences were already taught widely at universities, but clinical neuropsychology as a professional field was still in its infancy in Germany. One milestone in its development was the foundation in 1986 of the *Gesellschaft für Neuropsychologie* (Society for Neuropsychology; GNP) by a small group of psychologists. The number of members grew rapidly, and by 2020 had reached 1,600. The society has been issuing the journal *Zeitschrift für Neuropsychologie* since 1990. In 1993, the GNP introduced a certificate of qualification as a clinical neuropsychologist, although this initially did not confer state accreditation. In 1998, a paper jointly supported by one neurological (DGN), and three psychological societies (GNP, BDP, and DGPs; Gemeinsame Kommission Klinische Neuropsychologie, i.e., Joint Commission for Clinical Neuropsychology: K. Schoof-Tams, H. Flor, C. W. Wallesch, D. von Cramon, S. Gauggel, E. Kasten) optimized these regulations, which now takes place *via* a model that encompasses cooperation between university institutions specializing in neuropsychology and neuropsychological institutions.

## Health Insurance Cover for Neuropsychological Services

According to a nationwide overview (Membership Statistics GNP, 2018) a total of 1,129 neuropsychologists with the title of a “Clinical Neuropsychologist GNP” were certificated. The majority of GNP members (1,170 people, not all of them have completed training as clinical neuropsychologists) work in the inpatient and semi-inpatient sector, as, e.g., in clinical settings specializing in neurology, such as stroke units and neurological rehabilitation centers. In 2019 only approximately 210 clinical neuropsychologists worked in outpatient practices paid by the health insurance companies. As a result, patients with brain damage after accidents involving the CNS, strokes, tumor surgery or brain inflammation were well cared for in hospital and inpatient settings, but then had little opportunity to continue their neuropsychological treatment once at home. This often led to renewed deterioration in their capabilities and problems with returning to work. On the basis of evidence from hundreds of studies showing the effectiveness of neuropsychological therapy (review by Kasten et al., 2002), the *Gemeinsamer Bundesausschuss* (G-BA, i.e., Federal Joint Committee), the decision-making body that determines which medical treatments and procedures statutory health insurance will cover, decided in November 2011 that neuropsychological therapy should be included in what is called the outpatient ‘service catalogue’ of services covered by statutory health insurers. The *Kassenärztliche Vereinigung* (i.e., Association of Statutory Health Insurance Physicians) is responsible for approving the delivery and billing of outpatient neuropsychological diagnostic testing and treatment within statutory healthcare. Psychologists who wish to provide services in clinical neuropsychology must demonstrate, in particular, that they hold an additional neuropsychological qualification that essentially corresponds to the curriculum designed by the *Gemeinsame Kommission Klinische Neuropsychologie* (GKKN).

In order to understand this apparent complexity, it is useful to be aware of the strict separation in Germany between psychologists who work in hospitals/inpatient rehabilitation centers and those who practice in independent outpatient settings. Payment for the services provided by clinical neuropsychologists employed in neurological hospitals or clinics is paid for *via* a daily rate or *via* a flat rate per case. Since 2013 did it become permissible to bill outpatients’ therapy hours separately to health insurers *via* the *Kassenärztliche Vereinigung*. A further complicating factor is the coexistence in Germany of statutory and private health insurers. If a patient’s brain damage is the result of a work-related illness, the employer’s *Berufsgenossenschaft* (i.e., occupational insurance association) or the patient’s private accident insurance (if they have one), will pay. If neuropsychological treatment may enable the patient to return to work, Germany’s statutory pension insurance (“Deutsche Rentenversicherung”) system may sometimes cover the cost.

## Legal Basis

Legal basis of neuropsychological therapy is a decision of the German Federal Ministry of Health (Bundesministerium für Gesundheit) of November 24, 2011: *“Neuropsychological diagnostics and therapy serve to identify and treat brain-organic diseases, disorders of mental (cognitive) functions, the emotional experience, behavior and coping with the disease as well as associated disturbances in psychosocial relationships. The aim is to prevent emotional and motivational disorders after brain damage, to recognize following psychosocial impairments and activity restrictions of the patient and to heal or alleviate resulting pathological cognitive impairments. After inpatient acute or rehabilitation treatment, further timely outpatient treatment is desirable in case of persistence of aforementioned pathological disorders to improve the chances to achieve the greatest possible therapeutic success in disturbed higher brain functions. Already neuropsychological therapy is often used in these patients during the inpatient acute phase and can be continued on an outpatient basis.”*

According to this legal text, indications for neuropsychological therapy are:

“§4: […] e.g. *stroke or traumatic brain injury acquired brain damage or brain disease (organic brain disorder). The application of neuropsychological therapy is thereby only permissible in the case of pathological disorders in the following brain performance functions (partial performance areas): 1. Learning and memory, 2. Higher attentional performance, 3. Perception, spatial performance, 4. Thinking, planning and acting, 5. Mental disorders in organic disorders*.”

Paragraph §6 defines the qualifications of service providers: “*Authorized professionals are: Specialists in Neurology, Psychiatry, Psychotherapy, and Pediatric medicine with a focus on neuropaediatrics, neurosurgery and child and adolescent psychiatry. For neuropsychological diagnostics according to §5 and for neuropsychological therapy according to §7 are entitled: (1.) Specialists according to §1, (2.) medical psychotherapists with professional qualification in a procedure according to §13 of the psychotherapy guideline, (3.) Psychological psychotherapists with professional qualifications in a procedure according to §13 of the psychotherapy guideline, (4.) Child and adolescent psychotherapists with professional qualifications in a procedure §13 of the Psychotherapy Guideline. Each with an additional neuropsychological qualification with the same content or equivalent to the respective additional designation for Neuropsychology in accordance with the further training regulations of the state psychotherapist chambers or, if such is not the case, according to the model further training regulations of the Federal Chamber of Psychotherapists*.”

## The Treatment Process

Clinical neuropsychologists in hospitals primarily carry out diagnostic examinations with the patient and assess the severity of the problems they are experiencing. Clinical neuropsychology in an outpatient setting initially comprises five probatory sessions, then up to 60 sessions of 50 min each, which statutory insurance is required to cover, so that the therapist need only inform the insurer that they are taking place. Extensions beyond the 60th session are subject to application. Both individual and group therapy may take place, and family members or other caregivers can also be included (Bauer and Jürgensmeyer, 2021). The therapist initially conducts comprehensive diagnostic examinations of the patient’s cognitive functions. In Germany, clinical neuropsychologists also deliver therapy that, depending on the type of impairment the patient has, may seek to remove or compensate for it, but also take psychological problems into account and attempt to help the patient reintegrate into working and social life. The treatment takes place in close cooperation with specialists in neurology, general practitioners, physiotherapists, occupational therapists, speech therapists, social workers, and members of related professions. Specialist support services endeavor to enable patients to retrain or, in the case of young people, to engage in vocational training in occupational support schemes. Patients returning to work during or after treatment may do so *via* the ‘Hamburger model’, in which they begin by working a small number of hours on a few days of the week and then gradually increasing their hours until they have reached the upper limit of their capabilities. Patients who are unable to commence or return to mainstream work may join occupational schemes for people with disabilities or receive a disability pension. Neuropsychologists often take on a leading role in the planning and implementation of all stages of the treatment and reintegration process and are required, at regular intervals, to provide reports or comprehensive expert opinions on the patient’s progress and current state of health.

## Training as a Clinical Neuropsychologist

**Figure 2:**
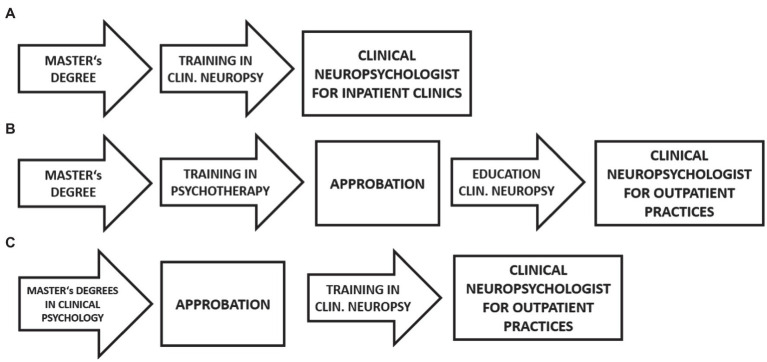
**(A)** Current training pathway for neuropsychologists in inpatient clinical settings. **(B)** Current training pathway for neuropsychologists working in their own practices. **(C)** Planned training pathway for clinical neuropsychologists working in their own practices. *Approbation* = state accreditation as a doctor or psychotherapist.

The training curriculum for neuropsychologists is currently changing. Thus far, psychologists have only been permitted to commence training in psychotherapy after having completed a degree in psychology. Changes to the German law on psychotherapists have transferred psychotherapy training, in part, to the Master’s degree in psychology (2 years education in Germany) which it is usual to complete after the Bachelor’s degree (3 years of education), i.e., a Master’s degree is an entry requirement for training in clinical neuropsychology (Figure 2).

In the current situation, qualifying as a neuropsychologist as the holder of a Master’s degree requires 3 years of clinical work in a recognized institution (or 2 years for psychologists who have already completed training in another branch of psychotherapy), 400 h of theoretical learning, 100 h of supervision, five written case reports, and an oral examination. Around 30 universities currently offer courses with an emphasis on clinical neuropsychology. Holders of Master’s degrees with neuropsychology as a focal area may have these attainments credited toward the theoretical content of the clinical neuropsychology curriculum. Theory lessons must be accepted for the training in clinical neuropsychology by the Gesellschaft für Neuropsychologie (GNP). These seminars must be accredited by the GNP, the inspection of the topic and content of a course is carried out by two independent experts. The recognition of non-GNP-accredited theory parts is possible, but requires an individual assessment with regard to equivalence. Previous scientific achievements, such as a dissertation in the field of clinical neuropsychology, may also be credited in the field of special neuropsychology.

A central component of the training is a clinical-practical activity under the guidance of an authorized clinical neuropsychologist. This institution must allow the candidate to enable the acquisition of extensive knowledge and skills in the treatment of patients suffering from organic brain disorders. Accreditation as a training institution for clinical neuropsychology is linked to proof of personnel, structural and organizational requirements that allow the teaching of treatment skills in accordance with the curriculum. Requirements are: (1) Certificate in Clinical Neuropsychology; (2) at least 5 years clinical-neuropsychological professional experience; (3) experiences as a lecturer for neuropsychological topics; (4) personal and professional suitability, and (5) continuous neuropsychological training of at least 100 h of training in the 5 years preceding the application (Source: GNP-Homepage).

Table 1 lists the subjects currently prescribed in the theoretical training, which are subject to continuous adaptation in line with neuroscientific advances.

**Table 1:** The German clinical neuropsychology training curriculum.

Foundational aspects of…	History + basics of neurosciences, neuroanatomy, neuroplasticity, psychopathology, psychopharmacology, conditions and disorders, courses of diseases, diagnosis, treatment, fields of work as a neuropsychologist, healthcare delivery systems, documentation, reports, quality assurance
Disorder-specific knowledge of…	perception, attention, memory, executive functions, language, arithmetic, motor skills, behavior, insight into illness
Care-specific knowledge of…	neuropsychology in childhood/adolescence, neuropsychology in older age, reintegration into work/school/social life, work as a neuropsychological expert (e.g., in court cases)

At the current time, clinical neuropsychologists only require *Approbation* (i.e., a ‘license to practice’ medicine, state accreditation as a doctor or psychotherapist) if they wish to work in their own outpatient practice.

The earnings of clinical neuropsychologists vary widely. According to information from the “Stepstone” job placement agency, the average annual salary of a neuropsychologist in Germany is between €41,300 and €58,500 (average: €47,500). The job agency “Karista” quotes a starting salary of between €2,000 and €3,000, which can be increased to up to €6,000 with increasing professional experience. The website “Gehalt.de” assumes €3,400 for professional newcomers of less than 3 years of experience and an increase to €5,400 for those professionals with more than 9 years of experience (Sources: https://www.stepstone.de/gehalt/Neuropsychologe-in.html, https://www.karista.de/berufe/neuropsychologe/lohn/ and https://www.lohn.de/beruf/neuropsychologe).

Self-employed neuropsychologists in their own practices can currently bill statutory health insurers for around €100.00 per 50 min.

## Comparison Between Germany and Other European Countries

According to studies by the Task Force Clinical Neuropsychology of the European Federation of Psychological Associations (EFPA), there are currently a lot of similarities, but also differences in the legal basis and the training of clinical neuropsychologists (see for example: Hokkanen et al., 2018, 2020; Kasten et al., 2021). As in Germany, almost all countries require a master’s degree as a minimum requirement (e.g., Austria, Belgium, Denmark, Estonia, England, Finland, France, Iceland, Luxembourg, Portugal, Switzerland, and Turkey). On the other hand, only a few countries have a law on the training of clinical neurologists (e.g., Austria, Italy, Netherlands, Poland, and Portugal). The duration of the training is about 3 years in Germany, internationally it varies between 12 months (e.g., Czech Republic) and 60 months (e.g., Norway). While education in Germany currently is still predominantly organized by a scientific society (GNP), in almost all other countries it is performed by universities (e.g., Belgium, Denmark, Estonia, Sweden, Switzerland, Russia, Portugal, and United Kingdom). The proportion of practical work during the training is defined differently and is between 5 months (Portugal) and 36 months (Germany) or between 500 h (Poland) and 3,600 h (Switzerland). The number of clinical neuropsychologists is about 1,200 in Germany, it varies between zero, e.g., in some east European countries (there were no neuropsychologists reported, e.g., from Bosnia, Serbia, or Bulgaria) and 2000 (e.g., Italy). France has a special position where every psychologist can also performe neuropsychology. While neuropsychologists in Germany do both, diagnostics and therapy, to about the same extent, there are many countries in which neuropsychologists mainly work diagnostically (e.g., Belgium, Denmark, Finland, Iceland, Italy, Luxembourg, Portugal, Russia, Spain, Sweden, and Turkey).

## Situation of Patient Care Delivered by Clinical Neuropsychologists in Germany

The group of patients requiring neuropsychological care in Germany comprises approximately half a million people who suffer a stroke every year, around 250,000 patients who have sustained traumatic brain injuries, and those requiring treatment for other conditions. Many of those with these conditions die, while others are only slightly injured, but around 800,000 people in Germany are living with the consequences of moderate damage to the CNS (quoted from Bauer and Jürgensmeyer, 2021).

Although Germany has a robust training system for neuropsychologists, there is a shortage of neuropsychologists for the outpatient sector. The estimated annual number of patients in Germany who could benefit from outpatient clinical neuropsychology is around 50,000 according to Kasten et al. (1997) and approximately 40,000–60,000 according to the *Gemeinsamer Bundesausschuss* (Federal Joint Committee, G-BA, cited by GNP, 2019). The G-BA places the number of full-time neuropsychologists necessary for delivering outpatient clinical care in Germany at about 1,100. The list of practitioners issued by the *Gesellschaft für Neuropsychologie*, 2019, however, currently contains only around 350 clinical neuropsychologists working in an outpatient setting. By contrast, in 2020 there were, for example, over 8,000 specialists in neurology and 12,000 psychiatrists practicing in outpatient settings (Statista, 2021).

The underlying problem is the requirement on neuropsychologists, in the last 20 years, first they have to complete training in a therapeutic modality approved by the state (i.e., psychoanalysis, cognitive behavioral therapy, or systemic therapy) before their training in clinical neuropsychology can attain recognition as an additional qualification. This extends the training period for neuropsychologists by at least 3 years. The *Gesellschaft für Neuropsychologie* (GNP) therefore has concerns about the care of patients suffering from disorders of cognitive capacity, emotional wellbeing and behavior after an organic brain disease. The chairman of the GNP (2020) has called for training as a specialist psychotherapist for clinical neuropsychology to follow immediately on from the general psychotherapy training path and *Approbation*.

## Limitations

Limitations of this article are that the state of education in Germany currently is in a change. While the training has so far been largely regulated by the GNP (Society for Neuropsychology) after the completion of the master’s degree, the focus is currently being placed more in the direction of training as a clinical psychologist during the master’s degree. Final information about future plans of how this training should ultimately look like is not yet possible.

## Author Contributions

The author confirms being the sole contributor of this work and has approved it for publication.

## Conflict of Interest

The author declares that the research was conducted in the absence of any commercial or financial relationships that could be construed as a potential conflict of interest.

## Publisher’s Note

All claims expressed in this article are solely those of the authors and do not necessarily represent those of their affiliated organizations, or those of the publisher, the editors and the reviewers. Any product that may be evaluated in this article, or claim that may be made by its manufacturer, is not guaranteed or endorsed by the publisher.
